# Void-Engineered Metamaterial Delay Line with Built-In Impedance Matching for Ultrasonic Applications

**DOI:** 10.3390/s24030995

**Published:** 2024-02-03

**Authors:** Rajendra P. Palanisamy, Luis A. Chavez, Raymond Castro, Alp T. Findikoglu

**Affiliations:** Materials Physics and Applications (MPA), Los Alamos National Laboratory, Los Alamos, NM 87545, USA; luis_chavez@lanl.gov (L.A.C.); raymondcas@lanl.gov (R.C.); findik@lanl.gov (A.T.F.)

**Keywords:** acoustic (ultrasonic) impedance, impedance matching, delay line, metamaterial, additive manufacturing (AM), concrete inspection

## Abstract

Metamaterials exhibit unique ultrasonic properties that are not always achievable with traditional materials. However, the structures and geometries needed to achieve such properties are often complex and difficult to obtain using common fabrication techniques. In the present research work, we report a novel metamaterial acoustic delay line with built-in impedance matching that is fabricated using a common 3D printer. Delay lines are commonly used in ultrasonic inspection when signals need to be separated in time for improved sensitivity. However, if the impedance of the delay line is not perfectly matched with those of both the sensor and the target medium, a strong standing wave develops in the delay line, leading to a lower energy transmission. The presented metamaterial delay line was designed to match the acoustic impedance at both the sensor and target medium interfaces. This was achieved by introducing graded engineered voids with different densities at both ends of the delay line. The measured impedances of the designed metamaterial samples show a good match with the theoretical predictions. The experimental test results with concrete samples show that the acoustic energy transmission is increased by 120% and the standing wave in the delay line is reduced by over a factor of 2 compared to a commercial delay line.

## 1. Introduction

Ultrasonic (acoustic) testing is a widely accepted nondestructive evaluation method. Ultrasonic inspection methods are generally used for flaw detection, material property identification, precise measurements, process monitoring, etc. [1,2,3]. Ultrasonic delay lines (sometimes also referred to as delay blocks or buffer rods) are used to delay the response that is typically used in the pulse-echo inspection mode, where echoes need to be separated temporally from large initial excitation pulse. Another very common application of delay lines (DLs) is in high temperature inspection where the sensor element cannot be attached directly to the target [4,5,6]. DLs are generally fabricated using a single material, and their impedance is matched only with the sensor, leading to a potentially large impedance mismatch between the target and DL. If the impedance mismatch is large, it causes a large amount of the acoustic energy to be reflected at the interface, and also creates a strong standing wave in the DL [7]. A single quarter wavelength matching (λ/4) layer is a popular solution to couple the source and target that has different impedance by taking advantage of the constructive and destructive interferences in the matching layer [8,9]. However, this method leads to a narrow operational frequency range [7]. Stacking multiple matching layers would theoretically improve the broadband characteristic of transmission, but it undesirably lengthens the coupling medium, especially for low-frequency (20–100 KHz) applications. Therefore, there is a need to develop novel DLs that provide a wide bandwidth (or, short pulse-width) impedance matching between the sensors and the interrogated structures.

A potential solution could be found in acoustic metamaterials. Acoustic metamaterials are artificial structures with unusual material properties that can control and manipulate acoustic signals. In general, most acoustic metamaterials consist of a periodic or aperiodic arrangement of small features, which overall behave like a continuous material with unconventional acoustic properties [10]. Metamaterials exhibit extraordinary acoustic properties, such as a negative refractive index [11], acoustic cloaking, and a local resonant structure [12,13,14]. One of the key drawbacks to metamaterials is the need for a high accuracy and precision in the fabrication of the oftentimes complex structures and geometries, which makes the use of common fabrication techniques time-consuming and costly [15]. Recent advances in additive manufacturing have greatly enhanced the ability to fabricate acoustic metamaterials [15,16]. The problem of acoustic impedance mismatch has also started to be addressed by various groups using acoustic metamaterials [17,18,19,20]. Research work by [7,21,22] showed that graded impedance matching metamaterials can improve broadband energy transmission in high frequency (1–5 MHz) applications.

Most existing state-of-the-art solutions involve complex metamaterial fabrication processes and are only focused on high-frequency applications. In this work, we developed a novel metamaterial DL that can be fabricated using a simple additive manufacturing (AM) process, which is particularly suited to long-wavelength, low-frequency applications, such as concrete or building material inspections. To accomplish a wide bandwidth (i.e., short pulse-width) performance, the impedance is gradually varied along the wave propagation direction, while matching the sensor impedance on one end and the interrogated structure on the other end. To determine the acoustic impedance of any material, the following two equations can be used. According to Equation (1), the acoustic impedance *Z* of a material is the product of its density ρ and acoustic bulk wave velocity *V*. Equation (2) shows how *V* depends on the elastic modulus *E* and Poisson’s ratio υ.

(1) Z=ρV

(2) $$V=\sqrt{\frac{E(1-\upsilon )}{\rho (1+\upsilon )(1-2\upsilon )}}$$

The idea of introducing cylindrical voids (much smaller than the propagating wavelength) in the bulk material changes some of the important mechanical properties, such as the effective density, elastic modulus, and Poisson’s ratio, which in turn change the effective acoustic impedance of the material. The choice of introducing cylindrical voids is only to ensure this is a simple additive manufacturing process. By carefully engineering the void size, shape, and distribution, it is possible to tune the impedance of the material. In this work, the relationship between the void density and the impedance of an additively manufactured material was studied experimentally, as well as theoretically using the Halpin–Tsai model [23,24]. Based on this combined empirical–theoretical analysis, along with the analysis of the accumulated printing errors, we designed a DL with gradually varying impedance values from about 6.3 MRayls to about 2.5 MRayls (impedance of the sensor). The performance of the fabricated metamaterial DL was compared to a commercially available uniform impedance DL. The experimental results show that the developed DL increased the energy transmission by 120% and reduced the strength of the standing wave by over a factor of two in the DL.

This manuscript is organized as follows: Section 2 describes the material and AM technique used to fabricate the void-engineered DL and discusses the error associated with printing. Section 3 shows the impedance measurement and the theoretical and empirical relationship between the void ratio and acoustic impedance of the printed samples. Further, based on the combined empirical–theoretical analysis, a metamaterial DL is designed and fabricated. In Section 4, the fabricated metamaterial DL and commercial DL are experimentally analyzed to compare their energy transmission and damage detection abilities. Finally, Section 5 summarizes the complete work and delivers the key conclusions of this research work.

## 2. Metamaterial Fabrication

**Figure 1:**
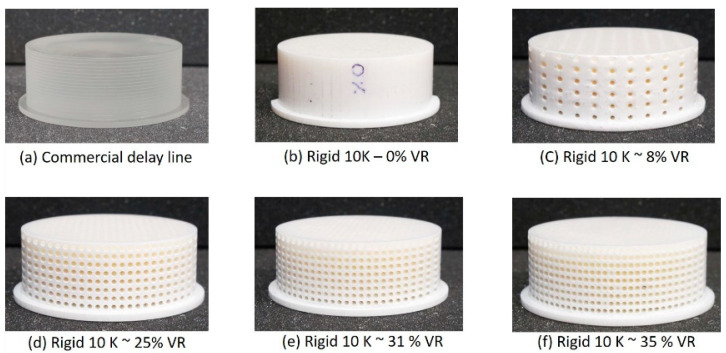
Commercial delay line (Ultran, Inc., State College, PA, USA) and 3D-printed metamaterial delay lines with different void ratios: (**a**) commercial delay line with a uniform acoustic impedance; (**b**–**f**) fabricated metamaterial delay lines with a measured void ratio between 0% and about 35%.

Metamaterial delay lines were fabricated using the stereolithographic additive manufacturing (SLA) method. A Form3+ SLA printer from Formlabs was used in this work. Because of its high stiffness, Rigid 10k resin was used as the base material to fabricate the samples. Preform, a software (version: V3.33.1) provided by Formlabs to convert design samples into 3D-printing instructions, was used to slice the samples as well as provide optimal printing parameters. To provide the most optimal printing parameters, Preform allowed for an adaptive printing speed that optimized each layer’s details. The standard parameters to create supports were provided by Preform and were left unchanged. These parameters provided a reproducibility standard to allow the samples to remain consistent while reproducing fine details. The printed samples were washed for 20 min using isopropyl alcohol, and ultraviolet curing was performed at 60 °C for 70 min, as suggested by the manufacturer. The samples were also polished using a wet sander tabletop at 600 grits, which allowed for an improved surface contact on the transmitting and receiving ends. For the purpose of this work, it was assumed that the mechanical properties of the feedstock materials were isotropic; however, it is important to note that the isotropicity of materials fabricated using SLA is unresolved [25]. The designed samples were fabricated with open paths for the voids, to allow for uncured resin to flow out during and after printing, as shown in Figure 1. The size of the fabricated voids had a direct impact in the broadband nature of the developed metamaterial DL; thus, different void diameters were tested. Also, void diameters smaller than 1.8 mm caused significant clogging with resin. Therefore, to achieve the best possible broadband frequency operation with minimal clogging, an optimal void diameter of 1.85 mm was chosen for all samples. Further, it was also noticed that cylindrical voids aligned in the direction of printing had clearer inner void surfaces compared to the voids aligned perpendicularly or at an angle to the printing direction. Thus, all the voids were fabricated with their alignment in the same direction, making the overall DL anisotropic in nature (refer to Figure 1).

**Figure 2:**
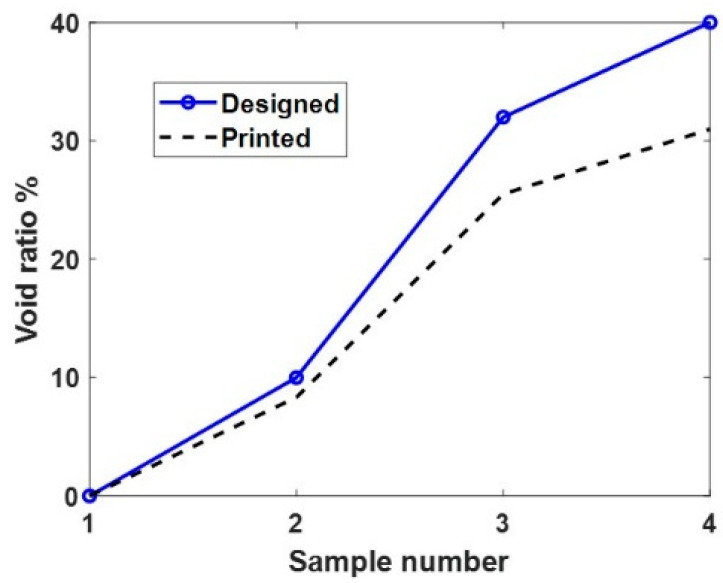
Designed vs. printed void ratios of the four samples.

In general, any feature or inclusions in the bulk material that are smaller than approximately one tenth of the wavelength (λ/10) of the acoustic signals are not expected to produce any significant reflections and, thus, are expected to behave as an effectively uniform material for the propagating wave. Therefore, for a given bulk wave velocity in the DL, the operating frequency should be chosen such that λ/10 is always greater than 1.85 mm (the designed void diameter). The commercial delay line was provided by Ultran, Inc, State College, PA, USA [26] for use with their sensor. As shown in Figure 1a, the commercial delay line had a uniform impedance of 2.64 MRayl, and it was cylindrically shaped with a diameter of 6.35 cm and a height of 2.54 cm. It also had surface features (grooves) to suppress side wall reflections to improve performance. The 3D-printed delay lines shown in Figure 1b–e had the same dimensions as those of the commercial delay line, with designed void ratios of 0, 10, 32, and 40%. The void ratio (VR) was calculated using Equation (3):

(3) $$VR=\frac{\rho_{r10k}-\rho_{DL}}{\rho_{r10k}}$$

where VR is the void ratio of the printed DL; $\rho_{r10k}$ is the density of the delay block with no voids, as shown in Figure 1b; and $\rho_{DL}$ is the density of the printed DL that is of interest. Due to discrepancies between the designed geometries and the fabricated samples, the actual VRs for the printed samples were 0, 8.33, 25.49, and 30.98%. Figure 2 shows the discrepancies between the designed and printed samples. It can be clearly seen that there is a cumulative error in printing as the VR increases. The knowledge of the printing error is used in Section 3.2 when designing the final metamaterial DL.

## 3. Theoretical and Empirical Analyses for the Design of the Metamaterial DL

In this section, the theoretical and empirical analyses of four initial samples were performed, based on the analyses and printing error observed in Section 2. A graded impedance metamaterial DL was designed and fabricated.

### 3.1. Impedance Measurement

**Figure 3:**
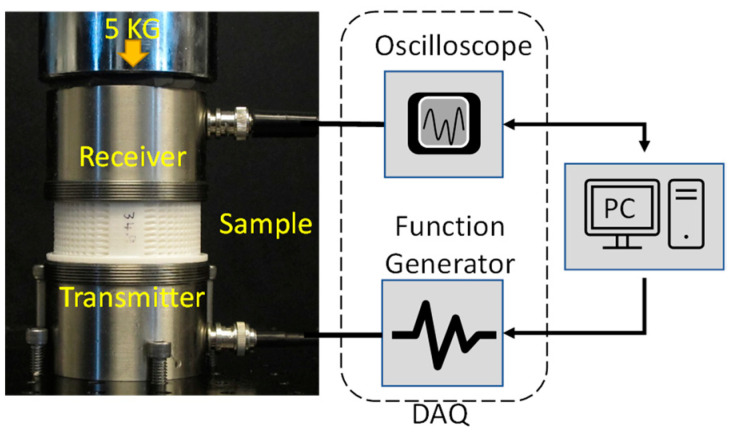
Experimental setup to measure the impedance of the 3D-printed samples.

**Figure 4:**
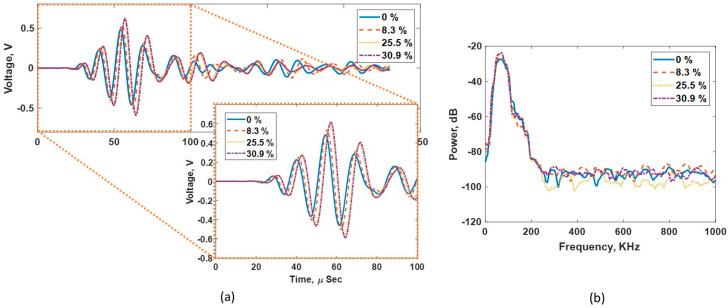
(**a**)Acquired time-domain signal showing the time delay as the VR increases. (**b**) Frequency power spectrum showing a consistent energy distribution for the samples with different VRs.

Measuring the impedance (*Z*) of the printed samples is very important to understand the acoustic behavior with different void ratios (*VRs*). The through-transmission experimental setup shown in Figure 3 was used to measure the velocity of the longitudinal or bulk acoustic wave in the samples. The density of each sample was calculated using the bulk volume and weight measurements. The impedance (*Z*) was calculated using the measured velocity (*V*) and density (ρ), as shown in Equation (1). The receiving and transmitting sensors used in the setup were from The Ultran, Inc., which had a center resonating frequency of 350 KHz (Model no: GRD350-D50). Data acquisition was performed using a Handyscope (model: HS5-540XMS-W5) from Tie-Pie engineering, Koperslagersstraat, The Netherlands. (A PC with the Python interface was used to control the DAQ and save the data). A Gaussian pulse with a center frequency of 100 KHz with 0.5 bandwidth (fractional bandwidth in the frequency domain of pulse) was used for all experiments. Glycerin was used as the couplant at the sample and transducer interface. A 5 kg dead load was applied on top of the receiver to ensure a good coupling and repeatable loading for all samples. The time of flight (TOF) values for the different samples were calculated using a cross-correlation function between the sensor–sensor and the acquired waveform from each sample. Figure 4a shows the acquired signal for the different samples. Observing the first arrived pulse within 100 µs, the longitudinal wave velocity is reduced as the VR in the sample increases. Figure 4b shows the frequency power spectrum for all acquired signals. A uniform energy distribution for samples with different VR is observed. This confirms that the selected frequency range (0–200 KHz) is not affected by introducing voids and/or their intensity.

Using the measured TOF and the length of the samples, the longitudinal velocity *V* in each sample was calculated and is listed in Table 1. The density ρ was calculated as the weight of the sample over the volume (excluding voids). Finally, the impedance *Z* of each sample was calculated using Equation (1). Based on the initial experimental observations, it is clear that the target impedance of the sensor (2.64 MRayls) is achieved with VRs below 0.4; thus, samples with higher VRs were not printed and studied in this paper.

### 3.2. Theoretical Verification of the Measured Impedances

**Figure 5:**
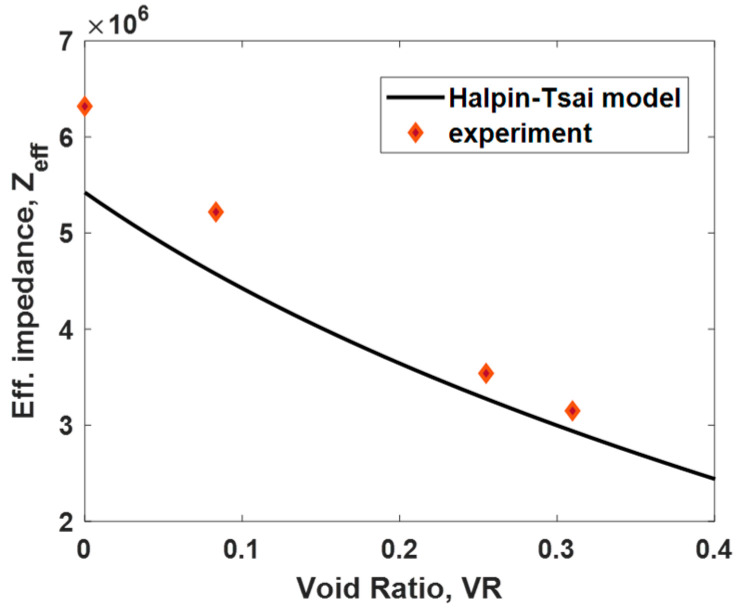
Comparison of the effective impedance calculated using the Halpin–Tsai model and the experimental measurement results at different void ratios.

Introducing voids in the bulk material, as shown in the previous section, is analogous to the case of unidirectional fiber-reinforced composite materials, where the fibers were replaced by voids in this study. Among the various composite approximation methods, the Halpin–Tsai model is popular due to its semi-empirical formulation and simplicity. In this study, we used the Halpin–Tsai model, as shown in Equation (4), to estimate the equivalent elastic modulus $E_{eff}$ in the direction of the wave propagation [23,24]. On the other hand, the effective density and Poisson’s ratio were calculated as described in Equations (6) and (7), respectively.

(4) $$E_{eff}=\frac{1+2\eta VR}{1-\eta VR}$$

where,

(5) $$\eta =\frac{\frac{E_{v}}{E_{m}}-1}{\frac{E_{v}}{E_{m}}+2}$$

(6) $$\rho_{eff}=\left(\rho_{v}\;VR\right)+\left(\rho_{m}\;\left(1-VR\right)\right)$$

(7) $$\upsilon_{eff}=\left(\upsilon_{v}\;VR\right)+\left(\upsilon_{m}\;\left(1-VR\right)\right)$$

where $E_{v}$ and $E_{m}$ are the elastic moduli of the void filler (air) and matrix (cured Rigid 10k resin), respectively; VR is the void ratio, as described in Equation (3); $\rho_{v}$ and $\rho_{m}$ are the density of the void filler and matrix, respectively; and $\upsilon_{v}$ and $\upsilon_{m}$ are the Poisson’s ratios. The approximate material properties of the void filler and matrix [27,28] are shown in Table 2. Using the effective material properties in Equations (1)–(3), the effective impedance, $Z_{eff}$, was calculated for various VRs, as shown in Figure 5. The model predictions and experimental results show a good match with some discrepancies at low VRs. These discrepancies could primarily be due to the approximate material properties used in the Halpin–Tsai model.

### 3.3. Design of the Metamaterial Delay Line

**Figure 6:**
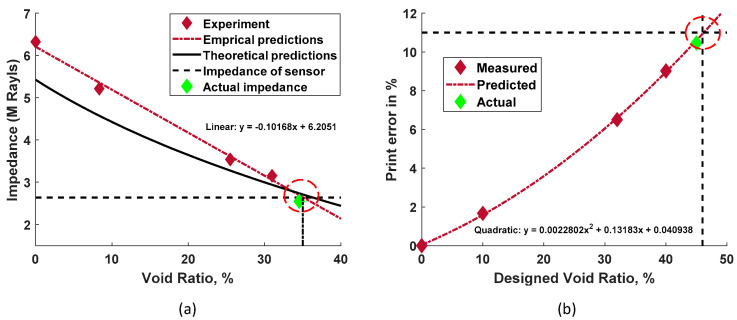
(**a**) Experimentally measured impedance of the printed samples at different void ratios, with the linear empirical impedance prediction (red dotted line) and theoretical Halpin–Tsai model prediction (black line). The impedance of the sensor is also included as a reference. (**b**) Accumulation of printing errors at different design void ratios, with the quadratic error prediction (red dotted line).

Figure 6a shows the change in impedance with the increase in the void ratio. Sample 1, representing the acoustic response of the pure Rigid 10K resin, has an impedance of 6.32 MRayl. With the increase in the void ratio, the impedance value decreases to 3.15 MRayl at a 30.98% VR, whereas the sensor and the commercial delay line have the same impedance of 2.64 MRayl. To achieve the best coupling, the impedance of the printed sample (DL) needs to be perfectly matched to that of the sensor. Using a linear curve fit over the measured impedance values and theoretical predictions, the predicted void ratio to achieve the optimal impedance match to the sensor was found to be about 35%, as shown in Figure 6a. The cumulative printing error described in Section 2 is plotted against the designed VR in Figure 6b. This figure also includes the quadratic curve fit over measured printing error to predict the printing error for the other void ratios. Based on the predictions in Figure 6b, a designed VR of 46% with a printing error of 11% was expected to provide an actual printed sample of 35% VR.

The fifth printed sample, shown in Figure 1f, exhibited a VR of 34.54%, very closely matching the required VR of 35%. The impedance of the fifth sample was measured using the same experimental setup and procedure discussed in Section 3.1, yielding an experimental impedance value of 2.55 MRayl (shown as a green diamond marker in Figure 6a), which closely matched the 2.64 MRayls impedance of the sensor. Thus, a delay line that was impedance-matched with the sensor was successfully fabricated using this 3D-printing approach. The designed and actual acoustic properties for all printed samples and the commercial delay line are listed in Table 1.

**Figure 7:**
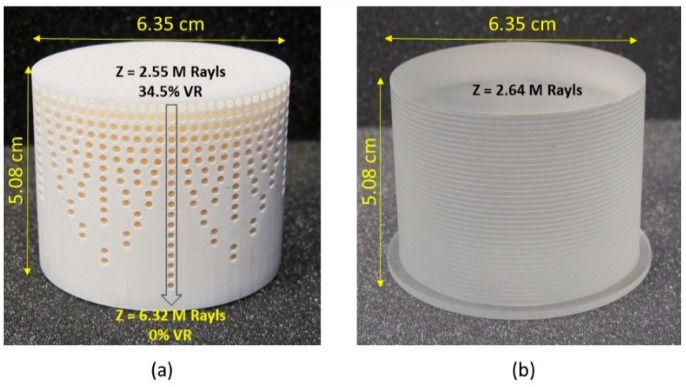
(**a**) Metamaterial delay line with varying acoustic impedance values. (**b**) Commercial delay line with a uniform acoustic impedance.

In order to obtain the best coupling between the sensor and the target medium, a delay line with a continuously varying impedance from 2.55 MRayls to 6.32 MRayls was designed, as shown in Figure 7a. Due to the graded impedance, the overall velocity of the longitudinal wave in the metamaterial DL was higher than that in the DL with a uniform impedance of 2.64 MRayls. The metamaterial DL had a cylindrical design with a diameter of 6.35 cm and a height of 5.08 cm. The void density was gradually reduced from 34.5% to 0 (column vice, with two rows having the same void density), as shown in the Figure 7a. Voids were equally distributed across the cross-section such that the propagating wave did not feel any local material change due to voids. It is important to note that the cylindrical void design, void diameter, and distribution were purely controlled and chosen to facilitate a simple additive manufacturing process. The appearance of the fabricated metamaterial DL was compared to that of the commercial DL, as shown in Figure 7b. The commercial DL had the same dimensions but a uniform acoustic impedance of 2.64 MRayls.

## 4. Experimental Validation

In this section, the performance of the commercial and metamaterial DLs were compared using a test involving concrete inspection. Experiments of through-transmission and pulse–echo configuration were conducted to evaluate the energy transmission efficiency and damage detection sensitivity, respectively.

### 4.1. Experimental Setup

**Figure 8:**
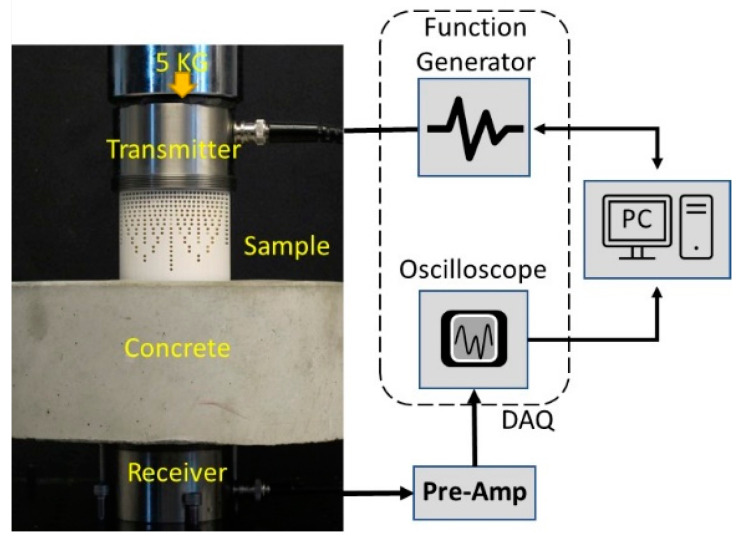
Experimental setup for the through-transmission measurement showing a schematic of the function generator, oscilloscope, pre-amp, and a laptop PC. Image with transducer, concrete, DL, and a dead load can be seen in the TT configuration.

**Figure 9:**
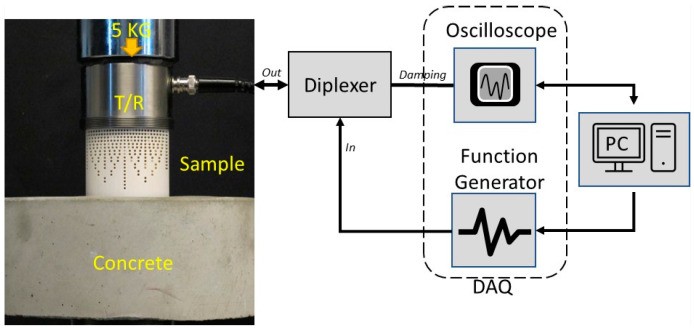
Schematic of the experimental setup for the pulse–echo measurement depicting a diplexer, function generator, oscilloscope, and a laptop PC. Image with transducer, concrete, DL, and a dead load can be seen in the PE configuration.

Figure 8 and Figure 9 show the experimental setup for through-transmission (TT) and pulse–echo (PE) concrete testing, respectively. The sensors and DAQ system used in this setup were the same as those in the experimental setup discussed in Section 3.1. In the TT setup, a pre-amplifier from Olympus, Tokyo, Japan (Model no: 5660B, with a custom frequency ranging from 20 KHz to 2 MHz), at a 40 db gain was used with the receiver to amplify the acquired signal. The concrete sample used for testing was cylindrical with a diameter of 21.6 cm and a height of 8.2 cm. The concrete sample had a density of 2242.5 kg/m^3^, longitudinal wave velocity of 3250 m/s, and an impedance of 7.3 MRayls. In the TT measurement, the transmitter generated an ultrasonic Gaussian pulse with a center frequency of 100 KHz with 0.5 bandwidth (fractional bandwidth in the frequency domain of the pulse). The generated stress waves passed through the delay line and the concrete sample before being acquired by the receiver (refer to Figure 8). A thin sheet of soft silicone (Ecoflex™ 00-10) was used as the couplant between the receiver and the concrete to increase the contact area. Glycerin was used as the couplant between the transmitter and the delay line interface. The couplant has its own impedance, which may favor either the commercial or metamaterial DL depending on their impedance value. Thus, to obtain a fair comparison between the commercial delay line and metamaterial delay line, there was no couplant used between the DL and concrete.

For the PE measurement, a Diplexer form RITEC, Inc., Warwick, RI, USA (Model No: RDX-6, with a custom frequency range of 10 kHz–30 MHz), was used to send and acquire the signal from a single transducer, as shown in Figure 9. The sensor, DAQ, and coupling conditions remained the same as those of the TT experimental setup. The metamaterial delay line was positioned in such a way that the circular surface with 2.55 MRayls was in contact with the transducer, and the circular surface with 6.32 MRayls was in contact with the concrete, as shown in Figure 8 and Figure 9. A 5 kg dead weight was applied on top of the transducer to ensure consistent coupling for all experiments. The experimental results for the PE and TT measurements using the commercial and metamaterial delay lines are compared and discussed in Section 4.2.

### 4.2. Results and Discussions

**Figure 10:**
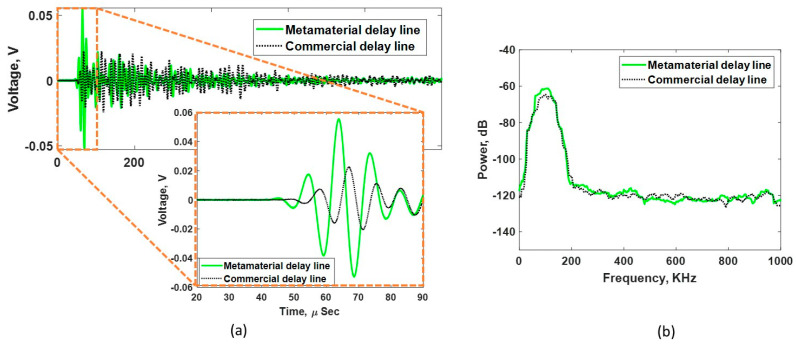
(**a**) Acquired waveform of the metamaterial and commercial delay lines during the through-transmission measurement. (**b**) Frequency power spectrum showing a similar energy distribution for both the DLs.

Figure 10a shows the complete time trace of the acquired signal along with a focus window for the first pulse within 90 µs using both DLs during the TT experiment. The amplitude of the first pulse received using the metamaterial DL is much higher compared to the commercial DL case. This increase in amplitude indicates a better energy transmission due to a better impedance matching. Since the impedance of the metamaterial DL is matched well with the sensor and also the concrete–receiver interface is unchanged, it can be assumed that the transmission loss across the transmitter DL and concrete–receiver interface remains the same for both the DLs during the TT experiment. Given this assumption, the only difference in transmission loss occurs at the DL–concrete interface. Equation (8) is used to calculate the energy transmission coefficient T at the DL–concrete interface for the commercial DL and metamaterial DL. The value of T for the commercial DL was 0.782 or −2.136 dB and for the metamaterial DL was 0.995 or −0.044dB. Thus, there was an expected overall 2.092 dB gain or 127.2% increase in signal.

(8) $$T=\frac{4Z_{1}Z_{2}}{{\left(Z_{1}+Z_{2}\right)}^{2}}$$

where T is the energy transmission coefficient, and $Z_{1}$ and $Z_{2}$ are the impedance of the selected DL at the DL–concrete interface and the impedance of concrete (7.3 MRayls), respectively.

The pulse from the metamaterial DL arrives earlier than that from the commercial DL, indicating an overall higher velocity in the metamaterial DL, as expected. Figure 10b shows the frequency power spectrum for both the DLs. The similar energy distribution indicates that there is no spectral filtering occurring in the fabricated DL. Further, the energy transferred by the metamaterial DL at the peak frequency is higher than that by the commercial DL.

**Figure 11:**
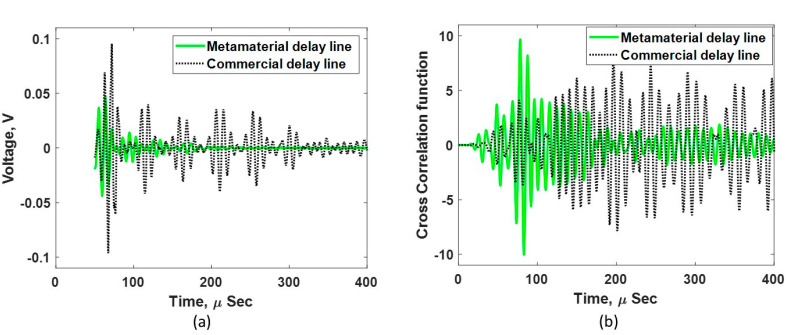
(**a**) Acquired waveform of the metamaterial and commercial delay lines using the pulse–echo measurement. (**b**) Processed data to identify the concrete end reflection.

**Figure 12:**
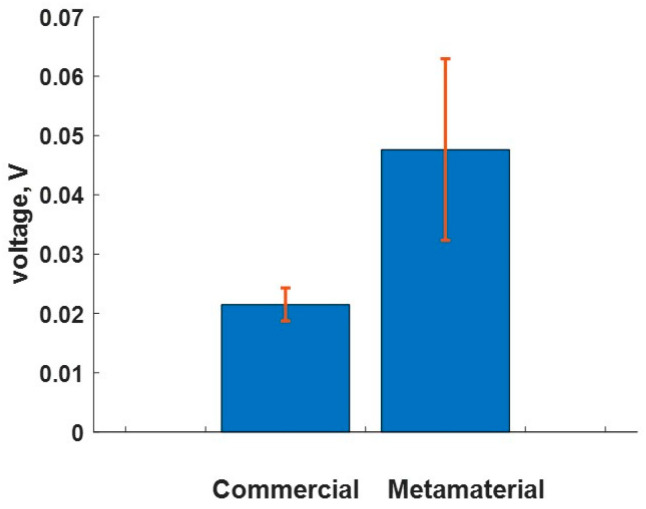
Comparison of the average voltage of the first pulse to arrive with the TT method.

For the metamaterial DL, the impedance at the sensor–DL interface is better matched compared to that at the DL–concrete interface. This poorer match at the DL–concrete interface is due to the maximum impedance limit of the Rigid 10K material at full density. However, the commercial DL has a larger impedance mismatch at the DL–concrete interface, leading to much of the energy being reflected at this interface. This phenomenon can be visualized more clearly in the PE measurement, as shown in Figure 11a. The TT measurement was repeated five times for each DL, and the statistical results of first pulse magnitude are shown in Figure 12. The average maximum voltage of the first pulse with the metamaterial DL was ~0.05 V, a 120% increase compared to the commercial delay line, which also matched closely with the expected 127.2% increase. A relatively large variation in the maximum voltage was noted for the metamaterial DL, which could be due to the inconsistent coupling at the DL–concrete interface that is associated with the unknown and non-uniform roughness of the printed metamaterial DL.

The acquired signals with the PE configuration for both the metamaterial and commercial DLs are shown in Figure 11a. The commercial DL exhibited a larger reflected signal, indicating a high impedance mismatch at the DL–concrete interface. Conversely, the reflected signal from the metamaterial DL was much lower, indicating that a better impedance match at the DL–concrete interface was achieved. These results are also in accordance with what was observed in the TT measurements, as shown in Figure 10. Similarly, the impedance mismatch led to a strong standing wave with a longer duration (about 380 µs) in the commercial DL. The standing wave for the metamaterial DL was much weaker and shorter (about 180 µs), as shown in Figure 11a. The characteristics of the standing waves in these DLs clearly indicate that the metamaterial DL is better suited for inspecting damage under these test conditions.

To better visualize the concrete reflection signal in the PE signal output in Figure 11a, the acquired signal was further processed, as follows. First, the transducer with the DL was detached from the concrete and a PE measurement was taken (while it was in the air). Then, the signals obtained while in contact with the concrete and air were subtracted. Finally, the resultant signal was cross-correlated with the input Gaussian pulse, resulting in the signal shown in Figure 11b. The concrete end reflection (at about 82 µs) can be clearly seen using the metamaterial DL, whereas the commercial delay line response was noisy and unable to detect the concrete end signal. This result, once again, indicates that the metamaterial DL has a superior energy transmission and damage detection capability for concrete inspections.

## 5. Summary, Conclusions, and Future Work

In this research work, we introduced the concept of void engineering using AM to tailor the acoustic properties of 3D-printed materials. Some of the key findings and conclusions are listed below.

- Acoustic DL samples with different, and graded, void ratios were successfully fabricated using additive manufacturing.
- The relationship between the void ratio and acoustic impedance was characterized, resulting in a metamaterial DL being designed and fabricated to achieve impedance matching both at the sensor and target interfaces.
- The experimental results, which are supported by the theoretical analysis, show the metamaterial DL has a 120% increased energy transmission and more than two-fold reduced standing wave ratio compared to the standard commercial DL; this enables its significantly improved damage detection capability.
- Our work establishes a promising pathway to control acoustic properties using practical metamaterials, which are fabricated using common additive manufacturing techniques, including achieving properties not readily available using natural materials.

In this research work, the application frequency and the range of tailorable impedance were restricted by the material properties of the resin and the resolution of the 3D printer. Our future work will focus on exploring other printing methods commercially available to fabricate structures with required finer features and materials. Such effort will potentially broaden the application areas of AM metamaterials to higher frequency and broadband acoustic applications. The effects of the void shape and distribution along with the statistical variability in the printed samples will also be explored as a part of our future work.

## Figures and Tables

**Table 1:** Designed, measured, and derived physical and acoustic properties of the commercial and printed delay lines.

Samples	Designed VR (%)	Printed VR (%)	Weight (g)	Density, ρ (Kg/M^3^)	Velocity, *V* (M/s)	Impedance, *Z* (MRayl)
Commercial Delay line	-	-	85.7	1070	2473	2.64
1	0	0	140.4	1750	3616	6.32
2	10	8.33	128.7	1600	3254	5.22
3	32	25.49	104.6	1300	2717	3.54
4	40	30.98	96.9	1200	2613	3.15
5	45	34.54	91.9	1140	2231	2.55

**Table 2:** Material properties of the void filler and matrix.

Material Properties	Void Filler (Air)	Matrix (Cured Rigid 10k Resin)
**Elastic modulus (E)**	0 GPA	10 GPA
**Poisson’s ratio (** υ **)**	0	0.36
**Density (** ρ **)**	1.293 kg/m^3^	1750 kg/m^3^

## Data Availability

Data are contained within the article.
